# Annual acknowledgement of manuscript reviewers

**DOI:** 10.1186/1471-2172-14-13

**Published:** 2013-04-10

**Affiliations:** 1 BioMed Central, Floor 6, 236 Gray's Inn Road, London WC1X 8HB, UK

## Abstract

**Contributing reviewers:**

The editors of BMC Immunology would like to thank all of our reviewers who have contributed to the journal in Volume 13 (2012).

## 

Soheila Ajdary

Iran

Lee-Ann Allen

USA

Karin Allenspach

UK

Stephen Anderson

USA

Mark Anderson

USA

Hideki Asanuma

Japan

Paul Ashwood

USA

Gunter Assmann

Germany

Deshratn Asthana

USA

Samantha Bailey-Bucktrout

USA

Troy Baldwin

Canada

Rolf Bambauer

Germany

Otmar Bayer

Germany

Davide Bedognetti

USA

Jimmy Berbée

Netherlands

Peter Beverley

UK

Andreas Beyerlein

Germany

Ulrich Blank

France

Dustin Blanton

USA

Damien Bresson

USA

Robert Bucki

USA

Georg Büldt

Germany

Margherita Cantorna

USA

Paul Carlson

USA

Filippo Castiglione

Italy

Edward Chan

USA

Robert Chevalier

USA

Fook Tim Chew

Singapore

Tian Chi

USA

Giulia Chinetti

France

Carlo Chizzolini

Switzerland

Sang Hyun Cho

South Korea

John Michael Conlon

United Arab Emirates

Gerardo Contreras

Mexico

Kevin Couper

UK

Michael Criscitiello

USA

Michael Croft

USA

Jane Dalton

UK

Toni Darville

USA

Jayasri Das Sarma

India

Hortensia De La Fuente

Spain

Michael Dean

USA

Gunnur Deniz

Turkey

Betty Diamond

USA

Mashenka Dimitrova

Bulgaria

David Dockrell

UK

Gert Doekes

Netherlands

Enrique Fernandez-Caldas

Spain

Gilberto Filaci

Italy

Thomas Forsthuber

USA

Keishi Fujio

Japan

Lisa Ganley-Leal

USA

Simani Gaseitsiwe

Botswana

Maria Cristina Gauzzi

Italy

Kenneth Gollob

Brazil

Jennifer Gommerman

Canada

Luis Graca

Portugal

Gregory Gromowski

USA

Eduardo Gudo

Mozambique

Francesca Gullo

UK

Ren Guocheng

China

Sonal Gupta

USA

Trond Halstensen

Norway

Matthew Hardman

UK

Guido Heine

Germany

Noriyasu Hirasawa

Japan

Sachiko Hirose

Japan

Christoph Holscher

Germany

Andreas Hutloff

Germany

Zsolt Illes

Hungary

Bruce Innis

USA

Dragana Jankovic

USA

Kyoung Yong Jeong

South Korea

Xiaoying Jiang

China

Vladimir Jurisic

Serbia

Naynesh Kamani

USA

Naoko Kanda

Japan

Marina Karakantza

Greece

Lavakumar Karyampudi

USA

Azad Kaushik

Canada

Kazuyoshi Kawakami

Japan

Can Kesmir

Netherlands

Yong-Sung Kim

South Korea

Haruki Komatsu

Japan

Frits Koning

Netherlands

Shyam Kottilil

USA

Damian Kovalovsky

USA

Gurumoorthy Krishnamoorthy

Germany

Gerald Krystal

Canada

Stefanie Kuerten

Germany

Yuki Kuwano

Japan

Miloslav Kverka

Czech Republic

Ming-Derg Lai

Taiwan

Maria Lampinen

Sweden

Mark Larche

Canada

Paul Lavender

UK

Dong Gun Lee

South Korea

Na Gyong Lee

South Korea

Daniel Legler

Switzerland

Helmar Lehmann

Germany

Agnes Lehuen

France

Bin Li

China

Min Li

China

Xuhang Li

USA

Yong Li

China

Shuen-Kuei Liao

Taiwan

Lina Lim

Singapore

Wenyu Lin

USA

Kuo Lin

Taiwan

Katri Lindfors

Finland

Zheng Liu

China

Fenyong Liu

USA

Aideen Long

Ireland

Liwei Lu

China

Fionnuala Lundy

UK

Holden Maecker

USA

Masoud Manjili

USA

Per Marits

Sweden

Ben Marsland

Switzerland

Luisa Martinez-Pomares

UK

Tomoji Mashimo

Japan

Paola Massari

USA

Mitsuru Matsumoto

Japan

Pamela Mccombe

Australia

Susan Mclennan

Australia

Kay Medina

USA

Henrik Mei

Ghana

Andrea Mencarelli

Italy

Laurence Morel

USA

Marija Mostarica-Stojkovic

Serbia

Susumu Nakae

Japan

Andreas Nandy

Germany

Yoshinori Naoe

Japan

Darren Newton

UK

Verena Niggli

Switzerland

Alistair Noble

UK

Masato Nose

Japan

Mairi Noverr

USA

Naganari Ohkura

Japan

Hajime Otani

Japan

Michael Otto

USA

Edgar Overton

USA

Philippe Pagni

USA

Josefin Paulsson

Sweden

Catherine Pellat-Deceunynck

France

Katarzyna Placek

USA

Diana Portale-Perez

Mexico

Alri Pretorius

South Africa

Troy Randall

USA

Alexandre Reis

Brazil

Rick Rest

USA

Anja Reutzel-Selke

Germany

Claude-Agnes Reynaud

France

Chae-Seo Rhee

South Korea

Benjamin Ricciardi

USA

Andrea Rinaldi

Italy

Fabricio Rios-Santos

Brazil

Craig Roberts

UK

Hosana Gomes Rodrigues

Brazil

Alan Rothman

USA

Martin Rottenberg

Sweden

Klemens Ruprecht

Germany

Ragnar Rylander

Sweden

Matías Sáenz-Cuesta

Spain

Bhaskar Saha

India

Pere Santamaria

Canada

Domenico Santoro

USA

Masanobu Satake

Japan

Thom Saunders

USA

Wilhelm Schoner

Germany

Danny Schust

USA

Marten Segelmark

Sweden

Rho Seong

South Korea

Alessandro Sette

USA

Judd Shellito

USA

Chao Shi

USA

Guangfeng Shi

China

Takeshi Shimosato

Japan

Sujay Singh

USA

Pejman Soroosh

USA

Hugo Soudeyns

Canada

Andreas Spittler

Austria

Philip Stahl

USA

Stefan Stevanovic

Germany

Alexandre Stewart

Canada

Phil Stumbles

Australia

Woong-Kyung Suh

Canada

Raquel Tarazona

Spain

Alberto Tedeschi

Italy

Friedrich Thaiss

Germany

Doug Thewke

USA

Wayne Thomas

Australia

Stephen Till

UK

Joerg Timm

Germany

Raul Torres

USA

David Tough

UK

Jean-Nicolas Tournier

France

Riccardo Troncone

Italy

Takeshi Tsubata

Japan

Gabriela Turk

Argentina

Benoit Van Den Eynde

Belgium

Mireille Van Gele

Belgium

Luc Van Kaer

USA

Mirinda Van Kleef

South Africa

Bernard Vanhove

France

Jose Luis Vela

USA

Subhash Verma

India

Audard Vincent

France

Eric Vivier

France

Shannon Wallet

USA

Peter Walzer

USA

Honghai Wang

China

Chyung-Ru Wang

USA

Owen Williams

UK

Emma Wilson

USA

Heather Wilson

UK

Cheryl Winkler

USA

Stefan Worgall

USA

Changyou Wu

China

Thomas Wynn

USA

Jianchuan Xia

China

Penghui Yang

China

Mahesh Yadav

USA

Felix Yarovinsky

USA

Ming Zeng

USA

Xing Zhang

USA

Guanglan Zhang

USA

Lu Zhang

China

Yingdong Zhao

USA

Jinfang Zhu

USA

